# Many Distinct Ways Lead to Drug Resistance in *BRAF*- and *NRAS*-Mutated Melanomas

**DOI:** 10.3390/life11050424

**Published:** 2021-05-05

**Authors:** Jiri Vachtenheim, Lubica Ondrušová

**Affiliations:** Department of Transcription and Cell Signaling, Institute of Medical Biochemistry and Laboratory Diagnostics, First Faculty of Medicine, Charles University Prague, Katerinska 32, 12108 Prague, Czech Republic; lubica.ondrusova@gmail.com

**Keywords:** melanoma, BRAF, NRAS, drug resistance, phenotype switching

## Abstract

Advanced melanoma is a relentless tumor with a high metastatic potential. The combat of melanoma by using the targeted therapy is impeded because several major driver mutations fuel its growth (predominantly *BRAF* and *NRAS*). Both these mutated oncogenes strongly activate the MAPK (MEK/ERK) pathway. Therefore, specific inhibitors of these oncoproteins or MAPK pathway components or their combination have been used for tumor eradication. After a good initial response, resistant cells develop almost universally and need the drug for further expansion. Multiple mechanisms, sometimes very distant from the MAPK pathway, are responsible for the development of resistance. Here, we review many of the mechanisms causing resistance and leading to the dismal final outcome of mutated *BRAF* and *NRAS* therapy. Very heterogeneous events lead to drug resistance. Due to this, each individual mechanism would be in fact needed to be determined for a personalized therapy to treat patients more efficiently and causally according to molecular findings. This procedure is practically impossible in the clinic. Other approaches are therefore needed, such as combined treatment with more drugs simultaneously from the beginning of the therapy. This could eradicate tumor cells more rapidly and greatly diminish the possibility of emerging mechanisms that allow the evolution of drug resistance.

## 1. Introduction

The incidence of melanoma has been slowly increasing worldwide in the last decades. Melanoma is derived from melanocytes, which originate from the neural crest precursor cells. Melanomas share many similar phenotypes with neural crest cell-derived melanocytes and express similar transcription factors [1]. A common problem in melanoma biology and therapy is tumor microheterogeneity. Different parts of a tumor possess distinct biological properties, which reflect the genetic and epigenetic diversity of the tumor cell subpopulations. These phenotypically distinct micropopulations also mirror the varying sensitivity to anticancer drugs. A hierarchy of tumoral cells exists in neoplasms. This is caused by both genetic and epigenetic mechanisms and results in a clonal evolution [2]. Driver mutations hit the genes critical for tumor progression and are considered the most important tumor promoters suitable for targeted therapy. Approximately 60% of melanomas harbor an activating *BRAF* (v-raf murine sarcoma viral oncogene homolog B1) mutation V600E (changing valine to glutamic acid and being highly predominant over other infrequent BRAF mutations). About 15% of melanomas contain *NRAS* (neuroblastoma rat sarcoma viral oncogene) mutations [3,4,5,6,7,8,9,10,11,12]. In mucosal melanomas, the *BRAF* and *NRAS* mutations are infrequent, which may reflect the lack of sunlight exposure [13]. Both mutational types are mutually exclusive, and each mutation substantially accelerates the activity of the MEK/ERK signaling pathway. It has long been known that *BRAF* mutation is already present in benign nevi, conferring the oncogene-induced senescence on nevus cells [14,15]. Given the importance of driver mutations, targeted drugs were developed to inhibit the aberrant activities of mutated *BRAF* and *NRAS* genes. Despite fundamental advances in the development of inhibitors focused on blocking the MAPK pathway in *BRAF*-mutated cancers, resistance remains a major hurdle to the enduring success of these therapies [11,16,17,18,19].

Large drug armamentarium exists to target mutated BRAF protein (dabrafenib, vemurafenib, encorafenib, sorafenib, GDC-0879, PLX8394) and the MEK/ERK pathway (MEK1/2 inhibitors: selumetinib, trametinib, binimetinib, cobimetinib; ERK1/2 inhibitors: ulixertinib, SCH772984, AZD0364, SCH772984). The number of the targeting NRAS-mutated drugs is limited, e.g., STK19(D89N) or pan-RAF kinase inhibitor belvarafenib). Many of these therapeutics have been already approved by the US Food and Drug Administration (FDA) to treat patients with advanced melanoma [20]. The inherited or acquired resistance appears almost invariably, typically months after the beginning of the treatment. Additionally, tumor cells can adapt to the administered drug and even require it for further proliferation. In such a case, the removal of the drug from the therapy is beneficial. This “drug holiday” [8,21,22,23] improves treatment, and melanoma cells can become senescent or undergo cell death [8]. Furthermore, a therapy-induced secretome that stimulates tumor growth has been described [24]. Here we describe some of the main molecular mechanisms underlying the resistance to mutated *BRAF* and *NRAS* genes in melanomas. Overcoming resistance is crucial for effective treatment and durable remission.

## 2. General Mechanisms Underlying Targeted Drug Resistance

The mitogen-activated protein kinase (MAPK) pathway is frequently hyperactivated, signaling a cascade in cancers, including melanomas. It is an essential pathway required for melanoma progression and the prime target for therapeutic intervention [22,25,26,27,28]. The MAPK pathway is activated by a broad spectrum of extracellular signals, including mitogens, growth factors, and cytokines. Besides mutations of the *BRAF* and *NRAS* genes, activating mutations have also been found in the downstream kinases MEK and ERK. MEK1 mutations can be associated with either BRAF or NRAS mutations, while ERK mutations are less frequent (reviewed in [29]). The *NRAS* and *RAF* genes (A, B, C) are the main activators of the pathway [30]). Mutated *CRAF* was shown to be a rare driver oncogene overexpressed in BRAF or NRAS melanoma cells. These cells were sensitive to CRAF, but not BRAF knockdown [31]. Although *BRAF* is the most frequently mutated driver gene in melanoma, *BRAF* mutations alone are nevertheless not sufficient for melanoma development. They are already found in benign nevi (see above).

Numerous reports revealed additional genetic alterations in various oncogenes and tumor suppressors beyond the *BRAF* and *NRAS* genes. They modulate progression into an invasive state and include *CDKN2A* (encoding the tumor suppressor p16 protein), *CDK4* (cyclin-dependent kinase 4), *PTEN* (phosphatase and tensin homolog), *NF1* (neurofibromin 1), *TP53*, *RAC1* (small GTPase 1), *MEK*, *MITF* (see below), *MC1R* (melanocortin 1 receptor), and many others [9,18] (reviewed also in [32,33,34]).

Although the main events leading to melanoma progression and drug resistance are genetic events (mainly *BRAF* and *NRAS* mutations), epigenetic mechanisms are also important. The early diversification of tumor micropopulations results in considerable heterogeneity of the whole tumoral population [35]. This results in different phenotypic features (some of the cells are *BRAFwt*, while some harbor the *BRAFV600E* mutation). Cancer heterogeneity is a common problem in oncology, hindering the more effective targeted therapy and considerably contributing to drug resistance [36,37]. Other factors, such as paracrine and autocrine cell communication and, e.g., the composition of stroma, can also promote tumor survival [38].

## 3. Resistance to Inhibitors of Mutated BRAF in Melanoma

### 3.1. BRAF and MAPK Cascade

**Figure 1:**
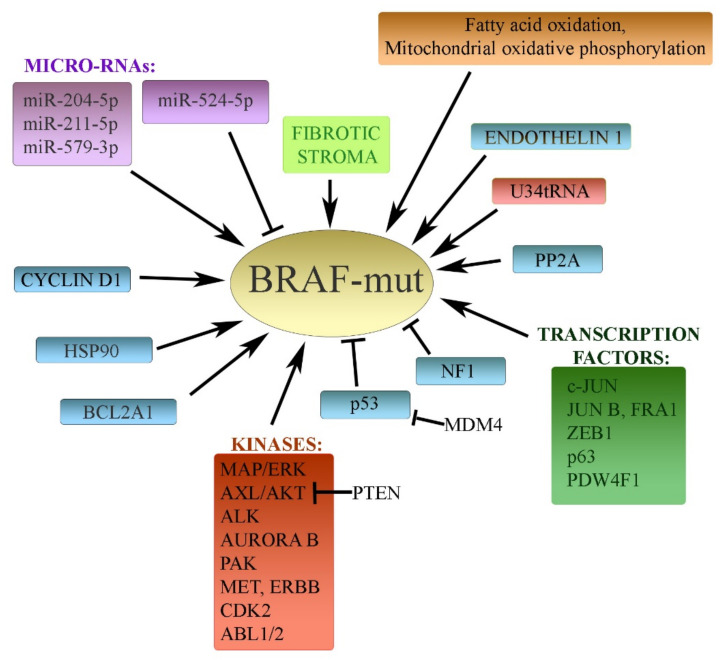
Some of the important mechanisms leading to resistance to mutated BRAF in melanoma. See text for a detailed explanation.

Wild-type BRAF (Figure 1) activates MAPK route upon upstream activation from NRAS through the formation of homodimers or heterodimers with CRAF. Mutated BRAFV600E does not form dimers and is capable of activating MEK kinase as a monomeric protein. BRAF inhibitors are inactive toward BRAFwt, but they effectively block the activity of monomeric BRAFV600E [39,40,41]. Interestingly, in BRAFwt cells, the expression of CRAF has been shown to be higher. Vemurafenib stabilized BRAF–CRAF heterodimers, thus helping to keep the MAPK pathway active [42]. The *BRAF* gene can be also amplified in a small subset of melanomas [40]. The BRAFV600E fusion protein AGAP3-BRAF has been reported to cause BRAFi resistance in melanoma [43]. The alternatively spliced variant of BRAFV600E has been shown to enhance the interaction with MEK. This depends on increased phosphorylation on MEKS729 and causes resistance in a subset of melanomas [44]. Among other very scarce BRAF mutations, a vemurafenib resistance-conferring, MEK/ERK-activating mutation of BRAF (BRAFL505H) has also been reported [45].

Resistance to BRAFV600E inhibitors logically comprises the biochemically closest downstream effectors in the MAPK pathway. Thus, MEK-activating mutations can bypass the block caused by inhibited mutated BRAF and reactivate the cascade [46,47]. Unfortunately, a resistance to the subsequently administered MEK inhibitor may also develop, causing the cells to become double-resistant. Reportedly, the ERK1/2 inhibitor SCH772984 efficiently suppressed the MAPK pathway and cell proliferation in BRAF, MEK, and combined BRAF/MEK inhibitor-resistant tumors [48,49]. However, long-term exposure of cells to the inhibitor also leads to acquired resistance through a mutation in ERK1 kinase [50].

### 3.2. Transcription Factors

Remarkably, resistance to the therapy is frequently mediated by mechanisms independent of ERK reactivation. A large number of mechanisms that may be very distant from the MAPK pathway can also cause BRAFV6000Ei resistance. Blocking these molecular events can release the sensitivity to the inhibitor. Alterations in other signaling pathways, transcription factors, oncogenes, and tumor suppressors are some examples of this kind of drug resistance.

Increased expression of the *c-JUN* oncogene, accompanied by the downregulation of SPROUTY4 and switching the phenotype to (EMT) epithelial-mesenchymal transition, was reported to be the reason behind BRAFi resistance in melanoma cells [51]. JNK/c-jun inhibitors synergy helped overcome BRAFi resistance [52]. Similar findings were reported by Titz et al., including co-targeting of BRAF and JUN which synergized in killing resistant cells [53]. In addition, JUN upregulation was a common outcome of BRAF inhibitor treatment in patients [53].

CRISPR–Cas9 knockout screen identified, besides ERK2 kinase, the transcription factors JUNB and FRA1 as essential factors responsible for the survival of BRAFi and combined BRAFi- and MEKi-resistant melanoma cells during drug holiday. The phenotype of resistant cells resembled the phenotype switch to EMT. The switch was ERK dependent, and the expression of the melanoma-specific transcription factor and oncogene MITF (melanoma-associated transcription factor) was decreased. Drug discontinuation synergized with the administration of dacarbazine, accompanied by decrease of MITF and its transcriptional target *BCL-2* [8]. In another study, JUNB and FRA1 transcripts were induced upon MAPKi withdrawal, and the total FOS protein level was increased [23]. Drug holiday, thus, may select against BRAFi/MAPKi-resistant melanoma cells. The involvement of JUNB and FRA1 level decrease, therefore, seems to be crucial for sensitizing cells to the “drug holiday treatment.”

Overexpression of the transcription factor POU4F1 (POU Class 4 Homeobox 1) contributed to the resistance to BRAFi in vemurafenib-treated melanoma cells by increasing MEK expression and activating MEK/ERK signaling. POU4F1 also increased the expression of MITF, further supporting resistance [54].

Furthermore, elevated levels of protein p63 were described in melanoma cells exposed to BRAF and MEK inhibitors. This was caused by its reduced degradation by the E3 ubiquitin ligase FBXW (F-box and WD repeat domain-containing), whose degradation is regulated by MDM2. FBXW was found to be downregulated in BRAF/MEKi-resistant cells. The MDM2 inhibitor Nutlin-3A restored FBXW7 expression and p63 degradation and resensitized resistant cells to the BRAFi inhibitor, thus underpinning the role of p63 in BRAFi melanoma resistance [12]. 

Zinc finger E-box-binding homeobox 1 (ZEB1) is a transcription factor known to induce phenotype switching, plasticity, and invasiveness. Consistently, high levels of ZEB1 expression were described to be associated with resistance to MEKi/BRAFi in BRAFV600E-melanoma cells. Conversely, ZEB1 inhibition promoted differentiation. It also inhibited tumor growth in vivo and sensitized melanoma cells to MEK/BRAF inhibitors, ultimately inducing cell death in resistant cells [55].

### 3.3. Protein Kinases

Many oncogenic protein kinases that act distantly from the MEK/ERK signaling have been demonstrated to cause resistance in BRAFi-treated melanoma cells. More pathways have cross-talks with MEK/ERK, and thus the hyperactivation of other oncogenic signaling cascades can often cause BRAFi resistance [56]. Studies have shown that the PI3K/AKT-mTOR pathway plays a role in the development of BRAFi resistance by triggering a surrogate survival signaling, leading to resistant BRAFi-treated melanoma cells [57,58]. The PI3K/AKT pathway has been recently identified as a key factor in stress-induced mutagenesis and stress-sensing mediating resistance in many types of cancer [59]. Inhibition of the PI3K/AKT may reactivate the sensitivity of melanoma cells to a BRAF inhibitor. Full activation of the PI3K/AKT pathway depends on the alterations and inactivity of its inhibitor and the tumor suppressor PTEN, which often regulates both acquired and intrinsic drug resistance. It was further shown that the oncogenic kinase AXL, together with AKT, mediates resistance to BRAFi, depending on the PTEN status. PTEN-inactive melanoma cells required only the active ERK pathway. However, AXL was shown to be an upstream effector of the AKT pathway-associated resistance to BRAFi in melanoma with wild-type PTEN [60]. Moreover, mutation of AKT kinase could alone trigger the adaptive resistance to BRAF inhibition [61].

Previous investigation also showed that the cell cycle regulator cyclin D1, often overexpressed in tumors, increased resistance concurrently with mutated or overexpressed CDK4 in BRAFi melanoma cells [62]. CDK2 also appeared to be a driver of resistance to inhibitors of BRAF and Hsp90 (XL888). Dinaciclib (a CDK2 inhibitor) attenuated resistance to both classes of inhibitors [63].

Oncogenic anaplastic lymphoma kinase (ALK) is an oncogenic driver in non-small-cell lung cancer and anaplastic large-cell lymphomas. Alterations in the *ALK* gene are gene fusions *EML4-ALK*, amplifications, or activating mutations. ALK was identified as another kinase conferring BRAFi resistance in melanomas. The PI3K/AKT signaling pathway, downstream of ALK, was required for ALK activity, and the ALK inhibitor ceritinib abrogated the BRAFi resistance in melanoma cells [64].

ABL kinase is frequently hyperactivated in tumors, e.g., in leukemias as a fusion gene with BCR. The expression of constitutively active ABL1/2 in melanomas has been described to be sufficient to promote resistance to vemurafenib, dabrafenib, MEK inhibitors, and their combination by inducing reactivation of MEK/ERK/MYC signaling. Targeting ABL kinases by the inhibitor nilotinib reversed the resistance and resensitized cells resistant to BRAF inhibitors. This indicates the role of ABL kinases as important factors supporting the active MEK/ERK pathway during the evolution of drug resistance in melanomas [65].

The P21-activated kinase (PAK) is a serine/threonine kinase mediating signaling in multiple pathways that are involved in numerous cell functions, including cell survival. This kinase has also been identified as one of the culprits causing BRAFi and combined inhibitor resistance in melanomas. Whereas the resistance to BRAFi was mediated classically through the MEK/ERK pathway reactivation (via the phosphorylation of CRAF and MEK), the resistance to combined treatment was independent of MEK/ERK activity. This second mechanisms involved-more complex mechanisms: regulation of JNK (c-Jun N-terminal kinase), β-catenin, and AKT/mTOR pathways. However, all types of resistant cells were sensitive to the PAK inhibitor PF-3758309. Thus, inhibiting PAK may also help treat patients whose tumors progress through initial inhibitor resistance in targeted therapies [66].

Aurora kinase B is overexpressed in melanoma. A study showed that the inhibitor of this kinase, HI-511, which also inhibits BRAFV600E, can suppress the growth of both vemurafenib-sensitive and vemurafenib-resistant melanoma cells. After inhibitor administration, both the MEK/ERK and PI3K/AKT pathways were inhibited. Aurora kinase B is thus another potential target in BRAFi-resistant cells [67]. 

Receptor tyrosine kinases from the MET and ERBB family have been reported to be involved in melanoma progression and metastasis, as well as in the development of therapy resistance. The epidermal growth factor receptor (EGFR), hepatocyte growth factor receptor (MET), and erb-b2 receptor tyrosine kinase 3 (ERBB3) were grossly overexpressed in BRAFi cells. Inhibition of these kinases with the combination of the drugs afatinib (an ERBB family inhibitor) and crizotinib (an MET inhibitor) proved to have deleterious and synergistic effects on melanomas. Interestingly, this effect was independent of their BRAF/NRAS mutational status [68].

### 3.4. MicroRNAs

Several microRNAs were described to be the cause of BRAFi resistance in melanomas. BRAFV600E-positive A375 melanoma cells were made resistant to vemurafenib, and two miRNAs, miR-204-5p and miR-211-5p, occurred overexpressed in these cells. Ectopic expression of both miRNA in parental and BRAFi-treated cells conferred vemurafenib resistance. Silencing their expression in BRAFi-resistant cells inhibited cell growth. The study thus shows that the miRNAs miR-204-5p and miR-211-5p enable resistance to BRAF inhibitors [69]. The miRNA miR-579-3p was shown to be a negative prognostic factor correlating with shorter survival, and its expression increased gradually during melanoma progression to later stages. The ectopic miR-579-3p maintained drug-resistant human melanoma cells, and its level sharply dropped in patients treated with targeted BRAFi therapies [70]. Conversely, the microRNA miR-524-5p, which behaves as a tumor suppressor in cancers, was indeed inhibitory for BRAFi-resistant melanoma cells. Overexpression of miR-524-5p reduced the proliferation and the migratory and invasive properties of BRAFi-resistant melanoma cells. In addition, the MEK/ERK signaling pathway was attenuated after treatment with miR-524-5p. This miRNA can therefore be considered a tumor suppressor for BRAFi-resistant melanomas [71].

### 3.5. Other Diverse Mechanisms

Additionally, very different mechanisms can contribute to BRAFi resistance. In earlier studies, the loss of the tumor suppressor NF1 has been observed in melanomas. Alterations of NF1 occurred in BRAF/NRASwt tumors, but also frequently occurred jointly with RAS or BRAF mutations. These BRAFV600E cells resulted in elevated resistance to RAF inhibitors. The loss of NF1 in mutated BRAF cells was therefore sufficient to overcome the upstream negative feedback and conferred resistance to vemurafenib. Furthermore, the MEK inhibitor trametinib attenuated MEK phosphorylation and exhibited greater potency than another MEK inhibitor, PD0325901, in NF1-null melanoma cells. Together, the loss of NF1 is associated with MEK dependence and resistance to BRAF inhibition [72]. 

The antiapoptotic BCL2 family member BCL2A1, a transcriptional target of MITF, is amplified in about one-third of melanomas. BCL2A1 expression is associated with more dismal clinical responses to BRAF inhibitors in melanoma patients. Cotreatment of melanomas with BRAF inhibitors and obatoclax, an inhibitor BCL2 family member, overcomes resistance to BRAF inhibitors in BCL2A1-amplified cells. Pharmacological inactivation of BCL2A1 thus leads to improved response to therapy by BRAF inhibitors [73]. 

Other MEK/ERK-independent mechanisms participate in the acquired BRAFi melanoma cell resistance. For instance, mRNA translation can have a key role in melanoma drug resistance. Wobble tRNA modifications are required for specific codon decoding during translation. It was demonstrated that BRAFV600E melanoma cells are dependent on U34 enzymes for survival. The wobble uridine 34 (U34) tRNA plays a key role in translation rewiring. It is induced by BRAFV600E and promotes glycolysis in cells through the regulation of the translation of HIF1α mRNA. U34 tRNA thus has an impact on BRAFi resistance in melanomas [74]. 

Recently, it has been described that melanoma cells can adapt to targeted therapies through a mechano-signaling loop involving autocrine remodeling. Melanoma cells secrete specific proteins to an extracellular matrix. In BRAFi-resistant cells, matrix remodeling and tumor stiffening were observed, depending largely on the Yes-associated protein (YAP) and myocardin. Targeting this biomechanical adaptation by Yes inhibitors may improve the sensitivity of BRAFi-resistant cells to targeted drugs [75]. 

Tumor stroma is known to be an important player in cancer progression. It has been reported that a more fibrotic stroma can influence the BRAFi resistance in melanomas [76]. Some studies support the notion that MEK/ERK inhibitor treatment in BRAF-mutant melanoma promotes the stromal microenvironment of melanoma cells toward a more fibrotic phenotype. Through this, drug resistance and tumor progression appears. Molecules such as fibronectin or type I collagen accumulate in the fibrotic stroma. Thus, therapeutically, relieving fibrosis in stroma with collagen or myofibroblast-targeting drugs could be promising. Such drugs can be pirfenidone, blocking myofibroblast proliferation, or aminopropionitrile, a lysyl oxidase inhibitor.

Another mode for overcoming BRAFV600Ei resistance is targeting the mitochondrial oxidative phosphorylation. IACS-010759 is a complex I mitochondrial oxidative phosphorylation inhibitor that inhibits the activity of both MEK/ERK and mTOR pathways. However, its activity is not synergized with MEK/ERK inhibitors. IACS-010759 enforces glycolysis and decreases nucleotide and amino acid pools. Thus, mitochondrial stress can mediate MEKi resistance and could be potentially clinically useful for melanoma treatment [77]. 

The authors of another study identified the fatty acid transporter CD36 as upregulated in MAPK-inhibited cells. These cells displayed increased fatty acid oxidation. Increased fatty acid oxidation was required for BRAFV600E-mutant melanoma cells to survive under the MAPK inhibition prior to acquiring drug resistance. Remarkably, the upregulation of CD36 itself was not involved in changes in fatty acid oxidation. These data indicated a potential metabolic approach to overcoming MAPKi resistance in BRAFV600E melanomas [78]. 

It is known that low-MITF/high-AXL expression signature is typical for invasive subpopulations of melanoma cells. Phenotype switching to this poorer phenotype is supported during the resistance to BRAF inhibitor therapy. It was shown that during the response phase of BRAFi therapy, endothelin 1 expression was increased. This occurred through the MITF-induced transcription and conferred drug resistance via MEK/ERK pathway re-activation. Antagonists of the endothelin receptor diminished AXL-high-expressing population and sensitized cells to BRAF inhibition. This suggests that targeting endothelin 1 can improve the BRAFi response and help overcome resistance [79]. 

## 4. Resistance to Inhibitors of NRAS Mutations in Melanoma

**Figure 2:**
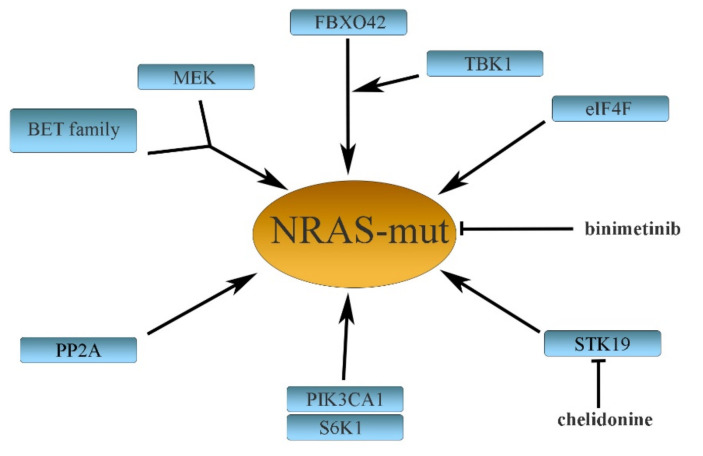
Some mechanisms causing resistance to mutated NRAS in melanoma. See the text for a more detailed explanation.

About one-third of cancers harbor mutations of *KRAS*, *HRAS*, or *NRAS* genes, occurring in codons 12, 13, and 61. In melanomas, only *NRAS* is exclusively mutated in about 15–20% of patients. The mutations are located predominantly in codon 61 (major mutations: Q61L, Q61K, and Q61R), while rare mutations are found in codons 12 or 13 [61,80,81]. Whereas many BRAFV600E-efficient inhibitors were found, specific NRAS inhibition is rather a disappointing matter compared with BRAF. The reason is that only a limited number of potentially active drugs have been developed [82,83]. Due to this, NRAS has been regarded as an “undruggable” target, and therapies aimed directly at NRAS mutations still remain elusive. BRAFV600E resistance develops during treatment and, similarly, resistance also appears when NRAS-mutant melanomas are treated with targeted drugs. Several distant mechanisms may be a cause of the acquired NRAS resistance (Figure 2).

Binimetinib (an MEK1/2 inhibitor) and the STK19 kinase inhibitor chelidonine were described to be relatively effective in NRAS-mutated melanomas [84,85], although controversies were raised against the efficacy of the STK19 inhibitor [86]. Like BRAF, NRAS also triggers the MEK/ERK signaling pathway. Therefore, binimetinib (used also in BRAFi-resistant melanomas) has been used for the treatment of NRAS-mutated melanomas either alone [85] or in combination with encorafenib [87]. Combining BET (bromodomain and extra-terminal domain) family and MEK inhibitors synergistically inhibited the growth of NRAS-mutant melanoma in mice not responding to MAPK inhibitors [82]. NRAS also stimulates the AKT/mTOR pathway, which is involved in mediating translational mechanisms. Thus, pharmacological targeting of the translation initiation complex component eIF4A, combined with an MEK inhibitor, seems to be an efficient treatment for NRAS-mutant melanomas [88]. 

Recently, Kelch domain-containing F-Box protein 42 (FBXO42) has been recognized as a factor causing the resistance of NRAS-mutant melanomas to trametinib. FBXO42 is involved in the transforming growth factor β-activated kinase 1 (TAK1) pathway, leading to the activation of p38 kinase. The TAK1 inhibitor takinib synergized with trametinib in eradicating NRAS-mutant melanoma cells [89]. 

A kinase TBK1 (TANK-binding kinase 1) is an atypical IκB kinase family member. It was demonstrated that NRAS overexpression increases TBK1 phosphorylation. Subsequent TBK1 downregulation inhibits cell migration and invasion, whereas its overexpression increases invasion of NRAS mutant melanoma cells [90,91]. TBK1 depletion cooperates with MEK inhibitors in the context of MEK-insensitive mutant NRAS, but not in wtNRAS. TKB1 thus appears to be a promising target in NRAS-mutant melanomas.

Another study revealed the E545K mutation in PIK3CA (a catalytic subunit of phosphatidylinositol 3 kinase PI3K) present in rare tumor subpopulations. These cells were nevertheless rapidly expanding during combined targeted therapy with MEK and CDK4 inhibitors. This represented a pivotal mechanism of resistance in NRAS-mutated melanomas. As PIK3CA-E545K increases S6K1 and S6 phosphorylation in the AKT/mTOR pathway, inhibition of the S6K1 kinase could be used to revert the resistance [92]. Growth inhibition of NRASQ61R melanomas (as well as BRAF-mutant melanomas) was also achieved by administering the protein phosphatase 2A (PP2A) inhibitor, a phendione derivative, or through depletion of PP2A catalytic subunits [26].

## 5. Discussion

Melanoma is a resilient kind of cancer and the deadliest form of skin cancer. Melanoma tumors harbor driver mutations mainly in two genes, *BRAF* and *NRAS*. Whereas many inhibitors directed to mutated BRAF and MEK have been synthesized and used for treatment, more specific and efficient NRAS-directed agents are still lacking. Resistance develops almost invariably during treatment with one inhibitor. In such setting, micropopulations of cells resistant to a drug gain growth advantage over others. This leads to the overgrowth of these cell populations and tumor resistance. Intratumoral heterogeneity is a driver of treatment resistance, causing phenotype switching and tumor plasticity [93,94,95]. Switching between various phenotypes, e.g., high MITF (less invasive and proliferating) and low MITF (slowly proliferating but invasive, giving rise to metastatic cells) is typical for melanoma. The extremely plastic phenotypic feature definitely underlies the highly malignant and metastatic properties of a melanoma. The plasticity is not necessarily a genomic event, but a consequence of epigenetic and environmental changes. It reflects, e.g., cell metabolic limitations [96,97], a stress-induced response of melanoma cells to environmental cues [98] or hypoxia [99]. Tumor plasticity is a pivotal mechanism in tumor resistance, appears as an adaptive plasticity to various epigenetic stimuli, and results in transcriptional reprogramming [100]. In an earlier study, the sensitivity of MAPK signaling to low-molecular drug inhibitors has been recognized in BRAF-mutated melanomas [101]. Vemurafenib resistance has been described. Resistant cells showed growing disadvantage upon drug withdrawal, which demonstrated the drug holiday phenomenon [102]. A typical mechanism of pigmentation in melanocytes is cAMP-dependent signaling. It supports the pathway leading to MITF stimulation through CREB. It is also noteworthy that it has been recognized as a mechanism conferring melanoma resistance to MAPK route inhibition [103]. Collectively, despite enormous effort, the exact molecular mechanisms why drug resistance evolves in mutated BRAF or NRAS melanomas remain elusive.

## 6. Conclusions

A number of pathways and, very surprisingly, MAPK-distant events have been known to contribute to melanoma development and progression, including the MAPK signaling pathway. This pathway notoriously overcomes its pharmacological inhibition and develops a remarkable trick to exploit the initially used drug for the further fitness and growth of malignant cells. The drug holiday becomes then a treatment possibility [8,23].

Sometimes, a combination with a second drug may help, especially when the two drugs have a synthetic lethality. Combined therapy of BRAF or NRAS inhibitors with various other agents (e.g., MEK/ERK pathway inhibitors) could be beneficial. Drug resistance is caused by adopting the phenotype plasticity and phenotype switching in tumor cells. Thus, the biological behavior of the tumor is constituted on the basis how rapidly the resistant cells evolve and ultimately exploit the drug to promote growth. A solution to overcome the acquired resistance could be, e.g., the use of three drugs simultaneously from the beginning of the treatment. This approach would profoundly decrease the probability that tumor cells will acquire resistance to all drugs. Additionally, the probability of a faster rate of tumor cell eradication by three efficient agents simultaneously is much higher. These drugs should target independent pathways important for melanoma progression. Another option is the use of agents that do not target the BRAF, NRAS, or MEK/ERK pathway at all, but inhibit essential mechanisms (such as antiapoptosis) important for tumor growth [104]. This approach can also constitute the basis for a more durable eradication of the tumor cells. In addition, such treatment can have an additional advantage, in that both BRAF/NRAS-mutated and non-mutated cells could be killed. A suitable cocktail of two—or better, three—drugs should be used that are effective outside pathways that notoriously acquire drug resistance.
